# A Prospective Study on Fertility Preservation in Prepubertal and Adolescent Girls Undergoing Hematological Stem Cell Transplantation

**DOI:** 10.3389/fonc.2021.692834

**Published:** 2021-06-30

**Authors:** Ida Wikander, Frida E. Lundberg, Hanna Nilsson, Birgit Borgström, Kenny A. Rodriguez-Wallberg

**Affiliations:** ^1^ Department of Reproductive Medicine, Division of Gynecology and Reproduction, Karolinska University Hospital, Stockholm, Sweden; ^2^ Department of Oncology-Pathology, Karolinska Institutet, Stockholm, Sweden

**Keywords:** fertility preservation, cryopreservation, ovarian tissue, infertility, hematological stem cell transplantation, chemotherapy, gonadal toxicity, oocytes

## Abstract

**Background:**

Hematological stem cell transplantation (HSCT) is an established method which has markedly increased the survival rate of hematologic malignancies since its introduction in the 1980’s. The conditioning for HSCT has known gonadotoxic effects and often leads to premature loss of fertility. In this study we have prospectively followed a cohort of girls undergoing HSCT and studied the outcomes of fertility preservation treatments performed before or after HSCT, as well as the long-term reproductive outcome.

**Methods:**

In this one-center prospective study, 39 girls counselled for fertility preservation prior to or after conditioning for HSCT for malignant or benign diseases at childhood or adolescence between 1990 and 2017 were included. The patients were presented with the option to undergo cryopreservation of ovarian tissue or oocytes depending on their age and the time available. Follicle counts of the ovarian tissue and number of oocytes collected before or after HSCT were compared between patients treated for benign and malignant diseases. Hormone measurements post HSCT treatment, including FSH and AMH, reproductive outcomes and overall survival until January 2021 were investigated.

**Results:**

In total, 34 girls and adolescents underwent fertility preservation before or after HSCT. Before HSCT, ovarian tissue was cryopreserved in 15 patients and two patients had oocytes preserved. Thirteen patients cryopreserved ovarian tissue after HSCT and seven patients returned to cryopreserve oocytes. Follicles were present in all tissue samples collected prior to HSCT, and in more than half of the samples collected post-HSCT. Half of the patients had spontaneous menarche or resumed menstruation post HSCT. Overall, 35 patients had survived at end of follow up and 7 patients had achieved parenthood.

**Conclusions:**

Since fertility loss is common following HSCT, fertility preservation should be offered to all patients. Fertility preservation treatments can be performed both before and after HSCT.

**Clinical Trial Registration:**

https://clinicaltrials.gov/show/NCT04602962, identifier NTC04602962

## Introduction

Survival of childhood cancer and severe anemias have improved over the past decades, in particular following the introduction of hematological stem cell transplantation (HSCT) in the 1980’s.

Today HSCT is a well-established and often curative treatment option for severe benign and malignant diseases. HSCT in patients with leukemia is performed in remission, which means that before HSCT the girls have already been treated according to different leukemia protocols for months or years. The chemotherapeutic conditioning prior to HSCT, sometimes combined with total body irradiation, is known to seriously damage the gonads, which causes endocrine dysfunction, gonadal insufficiency and confers a high risk of permanent infertility in both sexes (1). Gonadal toxicity of HSCT has also been reported in children (2–4). In girls the ovarian toxicity of HSCT may result in early impairment or absence of pubertal development and premature ovarian failure (2, 5). The risk of premature ovarian failure seems to increase with increased age at HSCT and is higher if alkylating drugs or total body irradiation are used (1, 2, 6–8). In general, the conditioning required for HSCT in patients with a malignant diagnosis requires more intense and more gonadotoxic treatment compared to that of patients with benign diagnosis, where the administered treatment regime does not have the same crucial impact on patient survival. Late effects of HSCT have prompted the introduction of reduced impact consolidation protocols, which have shown a positive effect on post treatment fertility when implemented in a young population (9, 10).

Current programs for fertility preservation have been developed at many centers worldwide. If patients are promptly referred, the counselling and performance of fertility preservation does not delay the initiation of a planned cancer treatment (11–13). Since future fertility is one of the most important issues raised by adolescent patients and emerging adult cancer survivors (14), pediatricians, specialists in reproductive endocrinology, oncology, surgery and reproductive medicine as well as skilled laboratory resources are all increasingly involved in the complex process of providing services for fertility preservation to young patients and children. The decision of how much time is available to undergo fertility preservation before starting chemotherapy for treatment of cancer should be discussed with the treating oncologist. In some cases it is better to postpone ovarian tissue harvesting until after the patient has received chemotherapy and has a better health status. Although collecting reproductive cells or tissue after chemotherapy treatment is not optimal (5), the abundant ovarian reserve in young girls may allow for this strategy (15).

In Sweden and the other Nordic countries, programs for fertility preservation are offered free of charge at tertiary care university hospitals to all patients facing treatments with risk of subsequent infertility (16). For pre-pubertal girls the only available option for fertility preservation is ovarian tissue cryopreservation (17). Post-pubertal girls have the additional option to cryopreserve oocytes, provided there is time for controlled ovarian stimulation and the acceptance of transvaginal follicle aspiration to retrieve the oocytes (13).

The Fertility Preservation Program at the Reproductive Medicine Clinic of Karolinska University Hospital was initiated in the 1970’s when methods for freezing sperm first became available. Since 1998 the program also included cryopreservation of embryos, ovarian tissue and thereafter oocytes, which may be elected by women, girls and transgender men before gonadotoxic treatments. All patients are currently followed as part of a long-term prospective observational study to evaluate the safety and efficacy of the treatments offered and results on this cohort have been reported (13, 18–21).

At present there are only a few studies reporting the long-term fertility and pregnancy outcome of girls undergoing fertility preservation before highly gonadotoxic chemotherapy due to severe benign or malignant disease (6, 13, 22, 23). In this study we report a prospective cohort of girls and adolescent women undergoing HSCT due to malignant and severe benign diseases, and the outcomes of fertility preservation treatments performed before or after HSCT, as well as the long-term reproductive outcomes and overall survival.

## Materials and Methods

### Data Source and Study Population

This is a single center, prospective study on fertility preservation and long-term outcomes in young girls and adolescents who have undergone HSCT. The cohort was diagnosed with malignant or severe benign disease between 1990 and 2017 at ages 0-19, and referred to the Pediatric Oncology-Endocrinology and Reproductive Medicine Center (Karolinska University Hospital, Stockholm) either before or after their HSCT. Patients were followed for reproductive outcomes and mortality until January 31^st^ 2021. All conditioning regimens for HSCT which included busulfan or melphalan, and regimens with high doses of other alkylating chemotherapy in combination with total body irradiation, were categorized as having a high risk of causing infertility. Conditioning using lower doses of alkylating chemotherapy with or without total body irradiation were categorized as intermediate risk treatments.

### Standardized Counselling of Girls and Teenagers

At the time of counselling, oral and written age adapted information on fertility preservation was provided by both a pediatrician and a specialist in reproductive medicine (13). The counselling of the patients and their families included information on available options for fertility preservation, as well as alternative ways of achieving future parenthood, such as egg donation or adoption. The possibility to return for fertility preservation after completed treatment was also offered.

### Fertility Preservation Methods

Ovarian tissue retrieval was planned and performed within a few days, and scheduled 1-2-weeks before beginning a conditioning regimen and HSCT whenever possible. The surgery was performed laparoscopically under general anesthesia, and usually planned simultaneously with other necessary procedures such as a central line insertion. Ovarian biopsies or unilateral oophorectomy was performed based on ovarian size and the treatment protocol. The ovarian cortex was sliced into small pieces (5 x 5-10 mm, with a thickness of about 1 mm) and cryopreserved by slow freezing or vitrification. In order to cryopreserve oocytes, ovarian stimulation and oocyte retrieval were presented as an option to adolescents post menarche. Ovarian stimulation with gonadotropins requires 1-2 weeks of gonadotropin stimulation prior to trans-vaginal follicle aspiration. The procedures for oocyte pick-up were performed under sedation and local anesthesia.

### Histopathology

Histopathological analysis of ovarian tissue was performed at the Department of Clinical Pathology and Cytology, Karolinska University hospital. One piece of the tissue was used to assess the presence of follicles and estimate the follicular density. Evaluation for presence of malignant cells was also requested.

### Patient Follow-Up

After completion of chemotherapy and HSCT, young pre-pubertal girls were followed at the Pediatric Oncology and Pediatric Endocrine units. Pubertal progression and hormonal levels were evaluated. Measurements of follicle stimulating hormone (FSH) and anti-Müllerian hormone (AMH) were performed at the Central Laboratory for Clinical Chemistry, Karolinska University Hospital. The pediatric endocrinologist was responsible for the initiation of puberty induction and hormone replacement during adolescence.

Patients were encouraged to return to the reproductive clinic after completed chemotherapy to evaluate their remaining ovarian reserve. If no fertility preservation was performed at the time of HSCT treatment due to lack of referral, time restraint or by patient’s choice, cryopreservation of ovarian tissue or oocytes was presented as an option post HSCT as long as some ovarian activity remained.

### Statistical Analyses

Outcomes of fertility preservation treatments were compared between patients with benign and malignant diagnosis, and between patients before and after HSCT. Pearson chi-square test was used for categorical variables, and Wilcoxon rank-sum test for continuous variables due to non-normal distributions.

Data management and analyzes were performed using Stata (StataCorp. 2019. Stata Statistical Software: Release 16: StataCorp LLC). All tests were two sided with a significance level of 5%.

## Results

The cohort included 39 female patients, aged 0-19 years at time of diagnosis, referred for fertility counselling between October 1998 and June 2020 (**Table 1**). The indication for HSCT was a malignant disease in 25 patients and severe hematologic disease in 14 patients. The most common malignant diagnoses were leukemia, lymphoma and sarcoma, while aplastic anemia and thalassemia were the most common benign diagnoses. Before HSCT, 21 patients with malignant disease underwent conditioning with a high risk of causing infertility and 4 patients received intermediate risk treatment. HSCT was given to 17 patients in first remission and 8 patients in second remission. For benign diseases, the conditioning regimen was classified as high risk in 6 patients and intermediate risk in 8 patients. After counselling, 34 of the 39 referred patients underwent fertility preservation. The five patients who did not undergo FP had received high risk conditioning for HSCT due to malignant disease in ages 0-4 years. They were counselled 9-17 years after HSCT, at which time they had high FSH (>20 IU/L) and undetectable AMH (<0.05 µg/L; one missing).

**Table 1:** Description of cohort.

Diagnosis	Benign disease n = 14		Malignant disease n = 25	
	No.	%	No.	%
Acute lymphocytic leukemia (ALL)			5	20.0
Acute myelogenous leukemia (AML)/Myelodysplastic syndrome (MDS)			7	28.0
Other leukemia			5	20.0
Lymphoma			4	16.0
Sarcoma			2	8.0
Neuroblastoma			2	8.0
Aplastic anemia	5	35.7		
Thalassemia major	3	21.4		
Amegakaryocytic thrombocytopenia	2	14.3		
Other hematological disease	3	21.4		
Other benign disease	1	7.1		
**Age at diagnosis**				
0-4	8	57.1	9	36.0
5-9	5	35.7	3	12.0
10-14	1	7.1	8	32.0
15-19	0	0.0	5	20.0
**Age at HSCT**				
0-4	4	28.6	7	28.0
5-9	4	28.6	5	20.0
10-14	4	28.6	6	24.0
15-22	2	14.3	7	28.0
**Year of HSCT**				
1990-1999	6	42.9	2	8.0
2000-2009	7	50.0	17	68.0
2010-2018	1	7.1	6	24.0
**Conditioning for HSCT**				
High risk of causing infertility	6	42.9	21	84.0
Intermediate risk of causing infertility	8	57.1	4	16.0
**First fertility preservation treatment**				
Before HSCT	4	28.6	13	52.0
After HSCT	10	71.4	7	28.0
Not yet performed	0	0.0	5	20.0

HSCT, Hematological stem cell transplantation.

### Ovarian Tissue Cryopreservation

Ovarian tissue cryopreservation was performed in 28 patients (**Table 2**). Among 16 patients with malignant disease, eleven were biopsied before HSCT, five were in second remission. The remaining five patients with malignant indications had undergone HSCT with high risk conditioning at the time of the biopsy. Eight of the 12 patients with benign disease had undergone HSCT at the time of the biopsy, whereof four had received high risk conditioning. The median age at ovarian tissue cryopreservation was 15 years in patients with benign disease and 13 years in patients with malignant disease. Ovarian biopsies of varying size were performed in 24 patients and unilateral oophorectomy was performed in 4 patients.

**Table 2:** Ovarian tissue cryopreservation by indication.

	Benign, n=12	Malignant, n=16	p-value*
**Age, median (range)**	15 (10-19)	13 (8-21)	0.242
**Follicles present, n (%)**			
Yes	8 (89%)	9 (75%)	0.422
No	1 (11%)	3 (25%)	
**Follicle density per mm^2^, median (range)**	25 (0-527)	49 (0-1519)	0.721

*Calculated using Chi-square test for proportion of samples containing follicles and Rank-sum test for age and follicle density. HSCT, Hematological stem cell transplantation.

Histopathological analysis of one piece of the ovarian tissue was performed in 21 samples. Of these samples, 11 were taken prior to HSCT and 10 post HSCT (**Table 3**). Follicles were found in 17 of the 21 samples examined. Follicles were found in all 11 analyzed samples taken prior to HSCT, and in 6 of 10 samples taken after HSCT (p=0.020). The density of follicles varied from 1 to 1519/mm^2^ in the samples containing follicles. There was no significant difference in median follicle density between samples from patients with benign and malignant disease (median 25 vs 49, p=0.721), or between samples taken before and after HSCT (median 56 vs 23, p=0.268). While the sample with the highest follicle density (1519/mm^2^) was taken after HSCT with high risk conditioning from a patient who was in second remission, the general trend was that samples taken after HSCT were less likely to contain any follicles (100% vs 60%, p=0.020). Among the patients with high risk conditioning for malignant disease who preserved ovarian tissue after HSCT, follicles were found in two of five analyzed samples. Among the eight patients with benign disease who cryopreserved ovarian tissue after HSCT follicles were found in four of five samples analyzed, three from patients who had undergone high risk conditioning and one after intermediate risk conditioning.

**Table 3:** Ovarian tissue cryopreservation before and after HSCT.

	Before HSCT, n=15	After HSCT, n=13	p-value*
**Age, median (range)**	13 (8-21)	14 (11-17)	0.471
**Follicles present, n (%)**			
Yes	11 (100%)	6 (60%)	0.020
No	0 (0%)	4 (40%)	
**Follicle density per mm^2^, median (range)**	56 (1-1384)	23 (0-1519)	0.268

*Calculated using Chi-square test for proportion of samples containing follicles and Rank-sum test for age and follicle density. HSCT, Hematological stem cell transplantation.

### Oocyte Cryopreservation

Two patients with malignant disease, and none with benign disease, received ovarian stimulation for oocyte cryopreservation before HSCT. After HSCT, seven patients have received ovarian stimulation for oocyte cryopreservation (**Table 4**). Three of these patients had benign disease, median FSH 5.4 (range 1.0-5.8), and four had malignant disease, median FSH 6.8 (range 2.2-13.0). Two of the patients with malignant disease had received high risk conditioning in second remission, two had intermediate risk treatment in first and second remission, respectively, while all three with benign disease had intermediate risk treatment. Three of these seven patients had previously cryopreserved ovarian tissue. All patients cryopreserved at least one oocyte (median 5, range 1-13) after one or two stimulation cycles. The median age at first oocyte cryopreservation was 20 years in patients with benign disease and 16 years in patients with malignant disease.

**Table 4:** Oocyte cryopreservation after HSCT, by indication.

	Benign, n = 3	Malignant, n = 4	p-value*
**Age, median (range)**	20 (17-26)	16 (14-20)	0.400
**Time since HSCT, median (range)**	11 (8-19)	8 (5-10)	0.229
**AMH, μg/L, median (range)**	0.62 (0.20-0.63)	0.85 (0.30-1.30)	0.629
**FSH, IU/L, median (range)**	5.4 (1.0-5.8)	6.8 (2.2-13.0)	0.229
**Number of oocytes, first stimulation, median (range)**	4 (3-7)	1 (0-12)	0.343

*Calculated using Rank-sum test. HSCT, Hematological stem cell transplantation.

### Long Term Follow-Up

The cohort has been followed a median of 17 years after HSCT (range 0-31 years). Four girls who had leukemia died of their disease; 0, 0, 4 and 9 years after HSCT. All of these girls had cryopreserved ovarian tissue and none had cryopreserved oocytes.

After HSCT, 11 of 14 patients (86%) treated for a benign disease and 6 of 21 patients (29%) with malignant diseases had spontaneous menarche or continued having menstrual periods (p=0.001). Premature ovarian failure post HSCT has occurred in 3 of 14 patients (21%) with benign disease and 15 of 21 patients (71%) with malignant disease (p=0.004). One patient resumed her periods but later during follow up experienced premature ovarian failure and data are missing for 4 patients.

At the end of follow-up (January 31^st^ 2021), 28 of the 34 patients who had cryopreserved ovarian tissue or oocytes were alive and at least 20 years of age (median 28, range 20-39). So far, seven patients have at least one child. The 21 adult women who did not yet have children at the end of follow-up were younger than the 7 women who had at least one child (median age 27 vs 31, p<0.001). None of the women that conceived had stored oocytes. None of the nine women with cryopreserved oocytes has yet returned for utilization.

Among the women with previous benign diagnoses, three conceived naturally and one using sperm insemination. Two of the women that conceived naturally had received high risk conditioning. Three patients of this cohort have re-transplanted ovarian tissue, all of whom had received high risk conditioning. All proceeded with ovarian stimulation, where two led to successful oocyte pickup and embryo transfer, and one woman treated with HSCT for a malignant disease in first remission has conceived. Additionally, two women treated for malignant diseases have achieved parenthood, one through oocyte donation and one has adopted a child. Both these women received high risk conditioning for HSCT in second remission.

FSH post HSCT was evaluated in 33 patients and the levels were significantly higher in the group treated for malignant disease than in the group treated for benign diseases (median 29.5 mIU/mL, range 2.2-127, and median 5.6 mIU/mL, range 1-58, respectively, p=0.016). AMH was measured in 23 patients post HSCT and had a median value of 0.5 μg/L (range 0-1.9) in the group treated for benign diseases and 0.08 μg/L (range 0-1.3) in the group treated for malignancy (p=0.235).

## Discussion

Our study on fertility preservation including the use of experimental methods such as cryopreservation of gonadal tissue for pre-pubertal and adolescent children of both sexes has been ongoing since 2002 (18). In this cohort 39 girls and teenagers undergoing HSCT for treatment of malignant or severe benign diseases received counselling on fertility preservation and 34 patients choose to proceed whereas five chose not to proceed with fertility preservation after counselling. The high level of participation among the included patients reflects the need for fertility counselling even at a young age. This is supported by studies showing that fertility is among the main concerns for young patients (24, 25). A total of 28 patients in the cohort cryopreserved ovarian tissue and nine could cryopreserve oocytes. Fertility preservation procedures could be performed before HSCT in 17 patients. The rate of fertility preservation after HSCT was 51% (20/39), this includes both patients who had not undergone previous fertility preservation procedures and patients who had previously cryopreserved ovarian tissue. An additional attempt at fertility preservation through oocyte cryopreservation can be of extra importance for patients with hematological malignancies where re-transplantation of tissue might reintroduce the malignancy.

Our results indicate that HSCT negatively impacts fertility in all patients, and more noticeably so in the patient group with malignant disease. Due to the small number of patients in our cohort who received total body irradiation, we were not able to assess the negative impact on fertility shown in previous studies (1, 2, 6–8). In the cohort of girls with a malignant disease we observed a significantly higher risk for premature ovarian failure and a lower chance of finding follicles in ovarian tissue retrieved after HSCT treatment. Adolescents who had resumed their menses could successfully undergo oocyte cryopreservation after HSCT. Among these patients, median AMH, number of oocytes retrieved and FSH was not significantly different between patients with malignant and benign disease, nor was FSH above the normal value for women in fertile age, which is in accordance with previous studies (26). However, FSH measured in all patients show the gonadotoxic effect of the treatments, visible especially among the patient group treated for malignant disease. It is also worth noting that, when looking at the full patient group with malignant disease, the measured AMH median lies well below the average for the age group, and median FSH well above (26, 27). These results are in line with the previously observed trend among women who returned after fertility preservation to attempt pregnancy, where a significantly lower live birth rate has been found among survivors of malignant disease when compared to women with a previous benign indication (6, 13, 22, 23).

The results suggests that referral to fertility counselling and treatment before HSCT is of outmost importance for patients undergoing HSCT. While oocyte cryopreservation might still be the preferred option for fertility preservation, cryopreservation of ovarian tissue is quickly becoming an established option for successful pregnancy and should be encouraged in young women and cases with time limitations (28). There have also been attempts to culture follicles *in vitro* to obtain mature oocytes from ovarian tissue with promising results (29).

This study is limited by the small size and the heterogeneity of the cohort. Although the study is prospective with long-term follow-up of the patients, information on fertility treatment attempts or live births occurring outside our center may have been missed. The age of the women with successful pregnancies is significantly higher than the mean age of the total cohort, which is lower than the mean age for first time mothers in Stockholm (30). In addition, considering the demanding treatments that the patients in the cohort have undergone, it is likely that the utilization rate will increase with longer follow-up. To better predict fertility after HSCT, additional factors such as the combined effects of age, treatment regime and individual trends in the oocyte reserve need to be explored in larger cohort studies.

## Conclusions

The results of this study underscore the need for fertility preservation in prepubertal and adolescent girls planned for HSCT treatment due to the gonadal toxicity inherent to the HSCT conditioning. Timely fertility counselling and the option of fertility preservation should be offered to all young female patients prior to or even after HSCT, whenever possible.

Today we lack tools to accurately predict which patients will lose their fertility, although patients with malignant diagnoses are at a greater risk compared to patients treated for benign diagnoses. Ovulation and fertility can be retained after HSCT but premature ovarian insufficiency early in life, before the patient plans to start a family, is a considerable risk. Our study shows that fertility preservation can be achieved before and also after HSCT and that these procedures enhance the chances of future fertility. Patients who have previously cryopreserved ovarian tissue may benefit from additional oocyte cryopreservation, as it reduces the chances of reintroducing malignancy through the transplant and the use of vitrified oocytes is now established at most reproductive centers. Fertility counselling and evaluation of the remaining fertility potential even after HSCT treatment in childhood can also provide an opportunity to undergo fertility preservation during adolescent years.

## Data Availability Statement

The original contributions presented in the study are included in the article, further inquiries can be directed to the corresponding author/s.

## Ethics Statement

Ethical approval for the study and for the follow-up of patients to adulthood was granted by the Ethical Review Board of Karolinska University Hospital (Dnr 427/03) and the Regional Ethics Committee of Stockholm (Dnr 2011/1158-31/2, 2014/470-32, 2016/2530-32 and 2018/2255-32). Written informed consent to participate in this study was provided by the participants’ legal guardian/next of kin.

## Author Contributions

KR-W designed the study and provided economic and administrative support. IW and KR-W collected the data. IW and FL analysed the data. IW, FL, HN, BB, and KR-W drafted and wrote the manuscript and revised the content. All authors contributed to the article and approved the submitted version.

## Funding

This study was funded by grants from The Swedish Childhood Cancer Fund (Grant number KP2016-0031, PR2016-0115), The Swedish Cancer Society (Grant number CAN 2017/704), Radiumhemmets Research Funds (Grant number 201313, 181272), the Stockholm county council (Grant number 20160626) and Karolinska Institutet (Grant number 2020-00339) (to KARW). The funding sources have played no role in the research design, analysis or reporting.

## Conflict of Interest

The authors declare that the research was conducted in the absence of any commercial or financial relationships that could be construed as a potential conflict of interest.

## References

[1] Donnez J Squifflet J Jadoul P Demylle D Cheron AC Van Langendonckt A. Pregnancy and Live Birth After Autotransplantation of Frozen-Thawed Ovarian Tissue in a Patient With Metastatic Disease Undergoing Chemotherapy and Hematopoietic Stem Cell Transplantation. Fertil Steril (2011) 95(5):1787.e1–4. 10.1016/j.fertnstert.2010.11.041 21145049

[2] Thibaud E Rodriguez-Macias K Trivin C Esperou H Michon J Brauner R. Ovarian Function After Bone Marrow Transplantation During Childhood. Bone Marrow Transplant (1998) 21(3):287–90. 10.1038/sj.bmt.1701075 9489652

[3] Cohen A Békássy AN Gaiero A Faraci M Zecca S Tichelli A. EBMT Paediatric and Late Effects Working Parties. Endocrinological Late Complications After Hematopoietic SCT in Children. Bone Marrow Transplant (2008) 41(Suppl 2):S43–8. 10.1038/bmt.2008.54 18545244

[4] Pfitzer C Orawa H Balcerek M Langer T Dirksen U Keslova P. Dynamics of Fertility Impairment and Recovery After Allogeneic Haematopoietic Stem Cell Transplantation in Childhood and Adolescence: Results From a Longitudinal Study. J Cancer Res Clin Oncol (2015) 141(1):135–42. 10.1007/s00432-014-1781-5 PMC11823712 25081929

[5] Biasin E Salvagno F Berger M Nesi F Quarello P Vassallo E. Ovarian Tissue Cryopreservation in Girls Undergoing Haematopoietic Stem Cell Transplant: Experience of a Single Centre. Bone Marrow Transplant (2015) 50(9):1206–11. 10.1038/bmt.2015.111 25961773

[6] Borgmann-Staudt A Rendtorff R Reinmuth S Hohmann C Keil T Schuster FR. Fertility After Allogeneic Haematopoietic Stem Cell Transplantation in Childhood and Adolescence. Bone Marrow Transplant (2012) 47(2):271–6. 10.1038/bmt.2011.78 21478918

[7] Vatanen A Wilhelmsson M Borgström B Gustafsson B Taskinen M Sarinen-Pihkala UM. Ovarian Function After Allogeneic Hematopoetic Stem Cell Transplantation in Childhood and Adolescence. Eur J Endocrinol (2014) 170:211–8. 10.1530/EJE-13-0694 24179099

[8] Jadoul P Anckaert E Dewandeleer A Steffens M Dolmans MM Vermylen C. Clinical and Biologic Evaluation of Ovarian Function in Women Treated by Bone Marrow Transplantation for Various Indications During Childhood or Adolescence. Fertil Steril (2011) 96(1):126–33.e3. 10.1016/j.fertnstert.2011.03.108 21550046

[9] Forgeard N Jestin M Vexiau D Chevillon F Ricadat E Peffault de Latour R. Sexuality- and Fertility-Related Issues in Women After Allogeneic Hematopoietic Stem Cell Transplantation. Transplant Cell Ther (2021) 27(5):432.e1–6. 10.1016/j.jtct.2021.02.003. 33789835

[10] Panasiuk A Nussey S Veys P Amrolia P Rao K Krawczuk-Rybak M. Gonadal Function and Fertility After Stem Cell Transplantation in Childhood: Comparison of a Reduced Intensity Conditioning Regimen Containing Melphalan With a Myeloablative Regimen Containing Busulfan. Br J Haematol (2015) 170(5):719–26. 10.1111/bjh.13497 25974284

[11] Rosendahl M Andersen CY Ernst E Westergaard LG Rasmussen PE Loft A. Ovarian Function After Removal of an Entire Ovary for Cryopreservation of Pieces of Cortex Prior to Gonadotoxic Treatment: A Follow-Up Study. Hum Reprod (2008) 23(11):2475–83. 10.1093/humrep/den248 18635528

[12] Kristensen SG Pors SE Poulsen LC Andersen ST Wakimoto Y Yding Andersen C. Time From Referral to Ovarian Tissue Cryopreservation in a Cohort of Danish Women. Acta Obstet Gynecol Scand (2019) 98(5):616–24. 10.1111/aogs.13575 30758835

[13] Rodriguez-Wallberg KA Marklund A Lundberg F Wikander I Milenkovic M Anastacio A. A Prospective Study of Women and Girls Undergoing Fertility Preservation Due to Oncologic and non-Oncologic Indications in Sweden-Trends in Patients' Choices and Benefit of the Chosen Methods After Long-Term Follow Up. Acta Obstet Gynecol Scand (2019) 98(5):604–15. 10.1111/aogs.13559 30723910

[14] Cherven B Meacham L Williamson Lewis R Klosky JL Gilleand Marchak J. Evaluation of the Modified Reproductive Concerns Scale Among Emerging Adult Cancer Survivors. J Adolesc Adult Oncol (2021). 10.1089/jayao.2020.0219. 33769891

[15] Rodriguez-Macias Wallberg KA Keros V Hovatta O. Clinical Aspects of Fertility Preservation in Female Patients. Pediatr Blood Cancer (2009) 53(2):254–60. 10.1002/pbc.21995 19340856

[16] Rodriguez-Wallberg KA Tanbo T Tinkanen H Thurin-Kjellberg A Nedstrand E Kitlinski M. Ovarian Tissue Cryopreservation and Transplantation Among Alternatives for Fertility Preservation in the Nordic Countries – Compilation of 20 Years of Academic Multicentre Experience. Acta Obstet Gynecol Scand (2016) 95(9):1015–26. 10.1111/aogs.12934 PMC5129549 27258933

[17] Mulder RL Font-Gonzalez A Hudson MM van Santen HM Loeffen EAH Burns KC. Fertility Preservation for Female Patients With Childhood, Adolescent, and Young Adult Cancer: Recommendations From the PanCareLIFE Consortium and the International Late Effects of Childhood Cancer Guideline Harmonization Group. Lancet Oncol (2021) 22(2):e45–56. 10.1016/S1470-2045(20)30594-5 33539753

[18] Borgström B Fridström M Gustafsson B Ljungman P Rodriguez-Wallberg KA. A Prospective Study on the Long-Term Outcome of Prepubertal and Pubertal Boys Undergoing Testicular Biopsy for Fertility Preservation Prior to Hematologic Stem Cell Transplantation. Pediatr Blood Cancer (2020) 67(9):e28507. 10.1002/pbc.28507 32649054

[19] Rodriguez-Wallberg KA Anastacio A Vonheim E Deen S Malmros J Borgström B. Fertility Preservation for Young Adults, Adolescents, and Children With Cancer. Ups J Med Sci (2020) 125(2):1–9. 10.1080/03009734.2020.1737601 32356507 PMC7721046

[20] Marklund A Eloranta S Wikander I Laczna Kitlinski M Lood M Nedstrand E. Efficacy and Safety of Controlled Ovarian Stimulation Using GnRH Antagonist Protocols for Emergency Fertility Preservation in Young Women With Breast Cancer—a Prospective Nationwide Swedish Multicenter Study. Hum Reprod (2020) 35(4):929–38. 10.1093/humrep/deaa029 PMC7192532 32313940

[21] Marklund A Lundberg FE Eloranta S Hedayati E Pettersson K Rodriguez-Wallberg KA. Reproductive Outcomes After Breast Cancer in Women With vs Without Fertility Preservation. JAMA Oncol (2021) 27(1):86–91. 10.1001/jamaoncol.2020.5957 PMC7677871 33211089

[22] Dalle JH Lucchini G Balduzzi A Ifversen M Jahnukainen K Macklon KT. State-Of-the-Art Fertility Preservation in Children and Adolescents Undergoing Haematopoietic Stem Cell Transplantation: A Report on the Expert Meeting of the Paediatric Diseases Working Party (PDWP) of the European Society for Blood and Marrow Transplantation (EBMT) in Baden, Austria, 29-30 September 2015. Bone Marrow Transplant (2017) 52(7):1029–35. 10.1038/bmt.2017.21 28287638

[23] Jensen AK Rechnitzer C Macklon KT Ifversen MR Birkebæk N Clausen N. Cryopreservation of Ovarian Tissue for Fertility Preservation in a Large Cohort of Young Girls: Focus on Pubertal Development. Hum Reprod (2017) 32(1):154–64. 10.1093/humrep/dew273 27816923

[24] Klosky JL Simmons JL Russell KM Foster RH Sabbatini GM Canavera KE. Fertility as a Priority Among at-Risk Adolescent Males Newly Diagnosed With Cancer and Their Parents. Support Care Cancer (2015) 23(2):333–41. 10.1007/s00520-014-2366-1 PMC4289648 25082365

[25] Young K Shliakhtsitsava K Natarajan L Myers E Dietz AC Gorman JR. Fertility Counseling Before Cancer Treatment and Subsequent Reproductive Concerns Among Female Adolescent and Young Adult Cancer Survivors. Cancer (2019) 125(6):980–9. 10.1002/cncr.31862 PMC6402962 30489638

[26] Fang T Su Z Wang L Yuan P Li R Ouyang N. Predictive Value of Age-Specific FSH Levels for IVF-ET Outcome in Women With Normal Ovarian Function. Reprod Biol Endocrinol (2015) 13:63. 10.1186/s12958-015-0056-6 26082101 PMC4470037

[27] Shebl O Ebner T Sir A Schreier-Lechner E Mayer RB Tews G. Age-Related Distribution of Basal Serum AMH Level in Women of Reproductive Age and a Presumably Healthy Cohort. Fertil Steril (2011) 95(2):832–4. 10.1016/j.fertnstert.2010.09.012 20970126

[28] Anderson RA Amant F Braat D D'Angelo A Chuva de Sousa Lopes SM Demeestere I. ESHRE Guideline: Female Fertility Preservation. Hum Reprod Open (2020) 2020(4):hoaa052. 10.1093/hropen/hoaa052 33225079 PMC7666361

[29] McLaughlin M Albertini DF Wallace WHB Anderson RA Telfer EE. Metaphase II Oocytes From Human Unilaminar Follicles Grown in a Multi-Step Culture System. Mol Hum Reprod (2018) 24(3):135–42. 10.1093/molehr/gay002 29390119

[30] Socialstyrelsen. Statistik Om Graviditeter, Förlossningar Och Nyfödda Barn 2019. Stockholm: The National Board of Health and Welfare (2020).

